# Histopathological changes in anatomical distribution of inflammatory bowel disease in children: a retrospective cohort study

**DOI:** 10.1186/1471-2431-12-162

**Published:** 2012-10-13

**Authors:** Jessica Tsang, Sheena Sikora, Donald Spady, Wael El-Matary

**Affiliations:** 1 Section of Pediatric Gastroenterology, Department of Pediatrics, Faculty of Medicine, University of Manitoba, Winnipeg, Manitoba, Canada; 2 Faculty of Medicine, University of Alberta, Edmonton, Canada; 3 Department of Pediatric Epidemiology and Research, Faculty of Medicine, University of Alberta, Edmonton, Canada

**Keywords:** IBD, Crohn’s, Ulcerative colitis, Children

## Abstract

**Background:**

Anatomical progression of pediatric inflammatory bowel disease is under-reported. The aim of this work was to examine possible changes in the anatomical distribution of IBD in pediatric patients at diagnosis and at follow up.

**Methods:**

In a retrospective cohort study, the medical records of children with inflammatory bowel disease were examined. Patients who had at least 2 endoscopic/colonoscopic examinations were included. Primary outcome was histopathological progression based on histopathological examination of biopsies taken during endoscopic and colonoscopic bowel examination. Factors predictive of disease progression were also examined.

**Results:**

A total of 98 patients fulfilled inclusion criteria (49 female, 54 with ulcerative colitis, range 2 – 17 years, mean age at diagnosis was 10.6 years, SD ± 3.67), the mean duration of follow up was 32.9 months (range 0.1 – 60 months, SD ± 8.54). In the ulcerative colitis group, 41% had disease progression and none of the examined variables (age, gender, laboratory markers, growth and disease activity at diagnosis) appeared to effect disease progression. In the Crohn’s disease group, 75% had disease progression. Girls (OR = 0.13, 95% CI 0.02 – 0.79) and patients with high erythrocytic sedimentation rate (OR=0.942, 95% CI 0.894 – 0.99) were predictive for disease progression.

**Conclusions:**

Despite maximum therapy, the majority of children with Crohn’s disease appeared to have histopathological disease progression. Female sex and high erythrocytic sedimentation rate seemed to be predictive for disease progression. None of the factors analyzed seemed predictive of disease progression in ulcerative colitis.

## Background

Inflammatory bowel diseases (IBD), mainly ulcerative colitis and Crohn’s disease are chronic, lifelong illnesses with young age of onset and a great potential for morbidity. The incidence of inflammatory bowel disease in the pediatric population is increasing [1,2]. About 20% of patients with IBD present before the age of 18 years old [1-7]. The natural history of these diseases is influenced by multiple factors of environmental and genetic origin. IBD showing colonic involvement has been reported to be more frequent in younger children when compared to older children [5]. Childhood-onset Crohn’s disease (CD) might reflect a more severe form of disease. Older patients with ulcerative colitis (UC) have demonstrated more cases of proctitis than younger patients with UC [5]. The current data have suggested that IBD may be phenotypically homogenous throughout childhood [8]. However, it is not clear if the histopathological distribution of IBD varies with time or not.

The aim of this study was to examine possible change in the histopathological distribution of IBD in pediatric patients at diagnosis and at follow up. While examining the anatomical distribution, the study also aimed to determine the possible variables that may be associated with or can predict change or progression in disease distribution.

## Methods

In a retrospective cohort study, the database of children with IBD at the Stollery Children’s Hospital, Edmonton, Canada and medical records of children with IBD who were 17 years of age or younger at diagnosis were examined. Patients with established clinical, endoscopic and radiological diagnosis of IBD, following recommendations of the North American Society for Pediatric Gastroenterology, Hepatology, and Nutrition [1], were included consecutively. All patients were diagnosed between January 2006 and December 2008.

The following variables were recorded:

1. Patient demographics (age at diagnosis, weight and height at diagnosis, gender, ethnicity and diagnosis)

2. Major presenting symptoms

3. Initial histopathological disease distribution at diagnostic endoscopy/colonoscopy

4. Subsequent histopathological disease distribution at follow up endoscopy/colonoscopy (primary outcome)

5. Initial and subsequent treatment

6. Activity indices using the Pediatric Ulcerative Colitis Activity Index (PUCAI) and the Pediatric Crohn’s Disease Activity Index (PCDAI) [9,10]

### Definition of disease progression

Disease progression was defined as a change in histopathological distribution of inflammatory bowel disease from diagnosis to follow up based on pathology of biopsies taken in each endoscopy and colonoscopy.

Routine biopsies during upper gastrointestinal endoscopy and colonoscopy were taken from lower esophagus, gastric antrum, upper duodenum, terminal ileum (TI), cecum, ascending colon, transverse colon, descending colon, sigmoid colon and rectum. Two biopsies were taken from each site. Successful intubation of TI was established in more than 90% of patients.

### Data collection

Patients eligible for the study were identified using the pediatric IBD database and data were collected using medical records. Inclusion criteria were children with established diagnosis of IBD who had endoscopy/colonoscopy both at diagnosis and at follow up. Patients who had one endoscopy/colonoscopy at diagnosis without further endoscopic follow-up were excluded. Those with incomplete charts/data were also excluded.

### Outcomes

The primary outcome was disease progression at the follow-up endoscopy/colonoscopy based on histopathology. Patients were then divided into two groups: those with disease progression and those with no disease progression. Secondary outcome was risk factors associated with disease progression.

### Statistics

Data were analyzed using Stata 9.1 ^(TM)^ (Data Analysis and Statistical Software, Texas, USA). Summary statistics were obtained and analyzed. Summaries (means, medians, ranges and standard deviations (SDs)) were obtained for continuous variables, while frequency distributions were provided for categorical variables. Cross-tabulations were done including all relevant variables against the outcome variable. Factors that may be associated with histopathological progression were explored using logistic regression analysis. These factors included age at diagnosis, gender, presenting symptoms, laboratory markers, growth parameters at diagnosis, and disease activity at diagnosis.

Results were considered statistically significant when *P* < 0.05.

### Ethics

The study protocol was approved by local health research ethics board,

## Results

During the study period, the medical records of 154 children with IBD (mean age 11.27+/−3.74 years, 83 boys, 74 with ulcerative colitis) were examined. Ninety eight patients fulfilled inclusion criteria (50 boys, range 2 – 17 years, mean age 10.6 years, SD ± 3.67); the mean duration of follow up was 62.9 months (range 0.1 – 60 months, SD ± 8.54). Of the 99 children with IBD, 54 had UC (54.5 %). In the UC group, 31 were females (57.4%); mean age of 9.9 years (SD ± 4.19)). In the Crohn’s disease group, 18 were females (40.1%); mean age was 11.2 years (SD ± 3.16).

### Patients with ulcerative colitis

**Figure 1:**
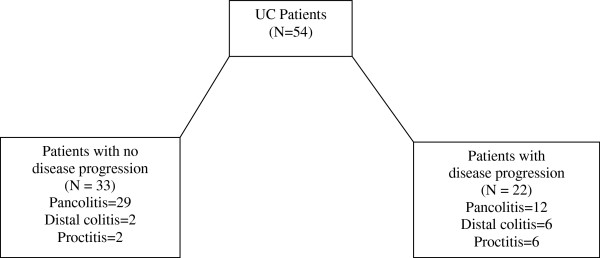
Initial histopathological disease distribution in children with ulcerative colitis.

Of the 54 children in the ulcerative colitis group, 22 (40.7%) showed change in disease distribution from diagnosis till the end of the follow-up period. The distribution of disease for patients with ulcerative colitis at diagnosis and at follow up is illustrated in Figure 1.

The most common disease distribution for the ulcerative colitis group was pancolitis (38/54, 70.4%), followed by left sided colitis extending to the splenic flexure (8/54, 14.8%) and distal proctitis (8/54, 14.8%). Eighteen patients (33.3%) showed evidence of disease. Of those showing disease progression, the most common distribution was pancolitis, left sided colitis and proctitis (6/18 each, 33.3% each). Of those presenting with left sided colitis, 3 (50%) went on to develop relative rectal sparing pancolitis, and 3 (50%) developed pancolitis at follow up. Of those presenting with distal proctitis, 4 progressed to left sided colitis (66.7%) extending to splenic flexure, and 2 developed pancolitis (33.3%) at follow up.

In patients with no disease progression, the most common distribution was pancolitis (32/36, 88.9%). Two (5.56%) had left sided colitis and 2 (5.56%) had distal proctitis.

Demographic, laboratory and clinical characteristics of children with ulcerative colitis are summarized in Table 1

**Table 1:** Mean values of demographic and laboratory data of pediatric ulcerative colitis patients

	**Progression Mean (SD)**	**No Progression Mean (SD)**	**p-value**
**Age at Diagnosis (years)**	9.36 (0.96)	9.99 (0.71)	0.99
**Duration of Symptoms Pre-Diagnosis (months)**	3.97 (0.95)	3.94 (1.08)	0.99
**Weight at Diagnosis (z-score)**	0.28 (0.38)	−0.32 (0.37)	0.48
**Height at Diagnosis (z-score)**	0.08 (0.33)	−0.37 (0.36)	0.37
**Albumin at Diagnosis (g/L)**	41.18 (1.10)	37.33 (1.60)	0.95
**ESR at diagnosis**	14.91 (3.89)	29.88 (5.25)	0.4
**AI at diagnosis**	39.05 (3.48)	46.67 (3.72)	1.000

ESR: erythrocytic sedimentation rate, AI: activity index.

Age at diagnosis, gender, presenting symptoms, laboratory markers, growth parameters at diagnosis, and disease activity at diagnosis were not significantly different between the two groups (disease progression vs. no disease progression). None of these variables was predictive of disease progression in the logistic regression model.

### Patients with Crohn’s disease

**Figure 2:**
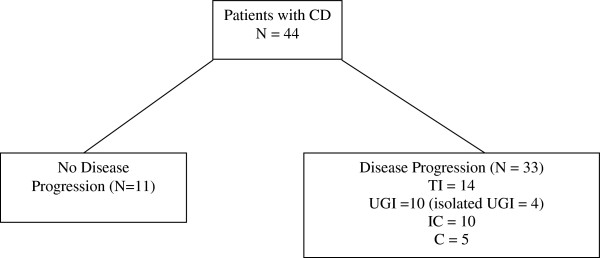
Initial histopathological disease distribution in children with Crohn’s disease.

Of the 44 children in the Crohn’s disease group, 33 (75%) patients showed disease progression from diagnosis till the end of the follow-up period. The distribution of disease at diagnosis and at follow up is illustrated in Figure 2.

The most common disease distribution for the CD group was terminal ileal (TI) disease (21/44 = 47.7%), followed by upper GI disease (duodenal/stomach/esophageal (UGI)) (17/44 = 38.6%), ileocecal/ileocolonic disease (12/44 = 27.3%), and isolated colonic disease (11/44 = 25.0%). Of note was that, there was an overlap of distribution in the UGI group and only 4 of them had an isolated UGI disease.

Thirty three patients showed evidence of disease progression (75%). Of those showing disease progression, the most common distribution to progress was TI involvement (14/33 = 42.4%). Eight out of 14 patients (57.1%) progressed to develop ileo-colonic (without rectal involvement) disease, 7 (50%) went on to develop upper gastrointestinal (UGI) disease, 4 (28.6%) went on to develop ileocecal disease.

In patients with no disease progression, the most common distributions were terminal ileal disease and upper GI disease, followed by colonic disease then ileocecal disease.

Demographic, laboratory and clinical characteristics of children with Crohn’s disease are summarized in Table 2.

**Table 2:** Mean values of demographic and laboratory data of pediatric Crohn’s disease patients

	**Disease Progression Mean (SD)**	**No Disease Progression Mean (SD)**	**p-value**
**Age (years)**	11.01 (0.60)	11.52 (0.58)	0.7
**Duration of Symptoms Before Diagnosis (months)**	6.77 (1.54)	8.83 (4.80)	0.83
**Weight (z-score)**	−0.80 (0.37)	0.05 (0.85)	0.28
**Height (z-score)**	−0.67 (0.35)	−0.29 (0.71)	0.06
**Albumin (g/L)**	35.14 (1.37)	35.88 (1.64)	0.32
**ESR**	35.61 (5.47)	27.50 (4.43)	0.02
**AI at diagnosis**	30.65 (2.59)	31.67 (2.04)	0.34

ESR: erythrocytic sedimentation rate, AI: activity index.

Using logistic regression analysis, ages at diagnosis, presenting symptoms and disease severity at diagnosis were not significantly different between the two groups. None of these variables was predictive of disease progression. However, it appears that there was a relationship between gender (OR = 0.13, 95% CI = 0.02 – 0.79) and disease progression. Disease progression was less likely in girls compared to boys. Moreover, ESR at diagnosis was significantly higher in those who had evidence of disease progression compared to those without disease progression and was predictive of disease progression (OR=0.94, 95% CI 0.90 – 0.99).

## Discussion

Our study examined histopathological changes in the distribution of IBD from diagnosis through follow-up in children and attempted to determine factors that would influence the progression of inflammatory bowel disease in this population.

The initial disease histopathological distribution in those ulcerative colitis patients with disease progression in our study was similar to those in previous studies, citing pancolitis, left sided colitis and proctitis as the most common distributions [1,3,4,7,8,11]. For the ulcerative colitis population, 40.7% of our patients showed disease progression. It appears that there is no relationship to age, duration of symptoms pre-diagnosis, weight, height, serum albumin levels, ESR and disease activity index at diagnosis. Interestingly, some patients with pancolitis in our series, who did not have any evidence of upper gastrointestinal involvement on initial endoscopy, had evidence of histopathological abnormalities on follow-up endoscopy. Upper gastrointestinal involvement in children with UC has been previously reported [12].

The most common initial disease histopathological involvements of Crohn’s disease in our study were terminal ileum, ileocecal and upper GI involvement. This finding agreed with previous studies which found that 50 – 70% of their pediatric CD patients had TI involvement at presentation [7,13,14]. About 75% of the CD patients in our study showed disease progression. It appears that there is a significant relationship between sex (OR = 0.13, 95% CI = 0.02 – 0.79) and disease progression. Based on our analysis, it appears that disease progression is less likely with those patients who are female when compared to those that are male. However, a previous study by Freeman demonstrated that CD tended to be more prominent in females when compared to their male counterparts [14]. Interestingly, in a previous study, it was determined that the switch to a female predominance began at around the age of adolescence [15]. It also appears that there is a significant relationship between ESR at diagnosis and disease progression in Crohn’s disease patients. Disease progression is more likely in those Crohn’s disease patients that have a higher ESR at diagnosis than those with a lower ESR at diagnosis. There are no previous studies to which we can compare these results.

Although we attempted to optimize the results obtained from our retrospective chart review, our study was limited in a few areas. Firstly, our study defined disease progression strictly as histopathological changes in disease distribution from diagnosis to follow up. Although this definition served the purpose of our study, perhaps future research could also look into disease progression in the sense of clinical presentation and symptoms, and whether or not factors affecting the clinical presentation and symptoms can predict IBD progression. Of note is that, large areas of small bowel could not normally be biopsied to explore histopathological involvement of Crohn’s disease.

Secondly, our study excluded those patients that did not have colonoscopy/endoscopy at follow up. Although that was necessary for the purpose of our study, perhaps future studies could look at disease status at follow up in another sense (to increase the number of IBD subjects available for the study). One can argue that patients with less controlled disease will be more likely to be scoped and hence selection bias is more likely to take place. Thirdly, for the purpose of our study, our subjects were divided into 2 main categories: disease progression and no disease progression. Future studies may benefit by further dividing the disease presentation and progression categories into mild, moderate and severe disease. In this sense, there may be a better understanding of which factors may affect certain stages of IBD in children.

## Conclusions

Despite maximum therapy, the majority of children with Crohn’s disease appeared to have disease progression. Female sex and high erythrocytic sedimentation rate seemed to be predictive for disease progression. None of the factors analyzed seemed predictive of disease progression in ulcerative colitis.

## Abbreviations

IBD: Inflammatory bowel disease; CD: Crohn’s disease; UC: Ulcerative colitis; ESR: Erythrocytic sedimentation rate; PCDAI: Pediatric Crohn’s disease activity index; PUCAI: Pediatric ulcerative colitis activity index; TI: Terminal ileum; UGI: Upper gastrointestinal.

## Competing interest

The authors declare that they have no competing interest.

## Authors' contribution

JT, SS and WE have made substantial contributions to conception, design and acquisition of data. JT and DS have made substantial contributions to analysis of data. JT and WE have been involved in drafting the manuscript and revising it critically. All authors have given final approval of the version to be published.

## Pre-publication history

The pre-publication history for this paper can be accessed here:

http://www.biomedcentral.com/1471-2431/12/162/prepub
